# Immunization with a DNA vaccine encoding *Toxoplasma gondii Superoxide dismutase* (TgSOD) induces partial immune protection against acute toxoplasmosis in BALB/c mice

**DOI:** 10.1186/s12879-017-2507-5

**Published:** 2017-06-07

**Authors:** Yuan Liu, Aiping Cao, Yawen Li, Xun Li, Hua Cong, Shenyi He, Huaiyu Zhou

**Affiliations:** 1 Department of Parasitology, School of Medicine, Shandong University, Jinan, Shandong Province, People’s Republic of China; 2 Department of Medicinal Chemistry, School of Pharmaceutical Sciences, Shandong University, Jinan, Shandong Province, People’s Republic of China; 3 Present address Department of Clinical Laboratory, The People’s Hospital of Rizhao, Rizhao, Shandong Province, People’s Republic of China

**Keywords:** *Toxoplasma gondii*, ME49 strain, *Superoxide dismutase*, DNA vaccine, BALB/c mice

## Abstract

**Background:**

*Toxoplasma gondii* (*T. gondii*) is an obligate intracellular protozoan parasite that infects all warm-blooded animals including humans and causes toxoplasmosis. An effective vaccine could be an ideal choice for preventing and controlling toxoplasmosis. *T. gondii Superoxide dismutase* (TgSOD) might participate in affecting the intracellular growth of both bradyzoite and tachyzoite forms. In the present study, the TgSOD gene was used to construct a DNA vaccine (pEGFP-SOD).

**Methods:**

TgSOD gene was amplified and inserted into eukaryotic vector pEGFP-C1 and formed the DNA vaccine pEGFP-SOD. Then the BALB/c mice were immunized intramuscularly with the DNA vaccine and those injected with pEGFP-C1, PBS or nothing were treated as controls. Four weeks after the last immunization, all mouse groups followed by challenging intraperitoneally with tachyzoites of *T. gondii* ME49 strain.

**Results:**

Results showed higher levels of total IgG, IgG2α in the sera and interferon gamma (IFN-γ) in the splenocytes from pEGFP-SOD inoculated mice than those unvaccinated, or inoculated with either empty plasmid vector or PBS. The proportions of CD4^+^ T cells and CD8^+^ T cells in the spleen from pEGFP-SOD inoculated mice were significantly (*p < 0.05*) increased compared to control groups. In addition, the survival time of mice immunized with pEGFP-SOD was significantly prolonged as compared to the controls (*p* < 0.05) although all the mice died.

**Conclusion:**

The present study revealed that the DNA vaccine triggered strong humoral and cellular immune responses, and aroused partial protective immunity against acute *T. gondii* infection in BALB/c mice. The collective data suggests the SOD may be a potential vaccine candidate for further development.

## Background

*Toxoplasma gondii* (*T. gondii*), one of the most successful parasites, is an obligate intracellular Apicomplexa protozoan, that can infect a wide variety of hosts including humans, livestock, and marine mammals and causes toxoplasmosis [1, 2]. *T. gondii* infection in immunocompetent individuals is clinically asymptomatic. However, toxoplasmosis occurred in immunocompromised individuals like those who are suffering from untreated HIV/AIDS is devastating [3]. Additionally, *T. gondii* infection not only causes abortions, stillbirths, and neonatal deaths in pregnant and lying-in women [4], but also leads to considerable economic losses in livestock, especially in sheep and goats [5, 6]. To make matters worse, humans are commonly infected by ingestion of raw or partly cooked meat containing *T. gondii* viable tissue cysts or by consumption of contaminated food and water with *T. gondii* oocysts [7, 8]. Despite several available chemical drugs, they are not absolutely safe with toxicities and have limited efficacy. In consideration of the serious impact of toxoplasmosis on the public health and economy, developing an effective vaccine against toxoplasmosis is of vital importance [9, 10].

Up to present, vaccines against toxoplasmosis have experienced inactivated or attenuated vaccine, subunit vaccine, multi-antigenic cocktails vaccine [11], but only one licensed vaccine based on the live attenuated S48 strain has been put into use in sheep, rather than human [12, 13]. DNA vaccine has been confirmed to elicit strong humoral and cellular immune responses and is more efficient and safer [14]. Thus, research priorities on prevention and treatment of toxoplasmosis could be shifted from chemical drugs, attenuated vaccine and subunit vaccine to DNA vaccines. In recent years, enormous achievements have been made on candidate antigens of DNA vaccines against toxoplasmosis [15].

*Superoxide dismutase* (SOD) is a major enzyme existing in a wide range of organisms, including animals, plants and microorganisms. SOD is mainly involved in the process of metabolizing O^2−^ in case of cell damage, namely, eliminating O^2−^ through conversing extra superoxide (O^2−^) anion into hydrogen peroxide and oxygen [16, 17]. This function of protecting cells from oxidative damages enables that SOD could be a potential research target in medicine, food industry and agriculture. Moreover, in *T. gondii*, several studies have shown that SOD might participate in affecting the intracellular growth of both bradyzoite and tachyzoite forms [18]. In our previous study, SOD gene was verified to have low sequence variation among ten examined isolates (RH, GT1, PTG, Prugniaud, CTG, TgCgCa1, MAS, TgCatBr5, TgCatBr64 and TgTouca), suggesting that SOD might be a potential vaccine candidate against *T. gondii* [19].

In this context, a DNA vaccine encoding *Superoxide dismutase* of *T. gondii* (TgSOD) was constructed, and then the murine immune responses and protection against challenge infection with the *T. gondii* ME49 strain were investigated.

## Methods

### Ethical statement

All animal care procedures were conducted in strict accordance with the Animal Ethics Procedures and Guidelines of the People’s Republic of China. The study was approved by the Institutional Animal Care and Use Committee of Shandong University under Contract LL2015–02, and all possible efforts were made to minimize distress on animals.

### Animals

Female BALB/c mice (6 weeks, 20+/−2 g body weight) were obtained from the Animal Centre of Shandong University (Jinan, China). All mice were raised under specific-pathogen-free conditions and fed with basal diet and water ad libitum. The infected mice were closely monitored twice daily for loss of appetite, dehydration, watery diarrhea, ruffled fur, lethargy and hunched posture. If obvious sufferings were observed, the mice were euthanized immediately by ether inhalation.

### Parasites and soluble tachyzoite antigen preparation

ME49 strain of *T. gondii* was kindly provided by Professor Quan Liu, Department of Parasitology, Military Veterinary Institute, Academy of Military Medical Sciences, China. The parasite was grown in human foreskin fibroblast (HFF) cells and maintained in complete medium supplemented with 10% fetal calf serum, 25 μg/ml gentamycine and 2 mM glutamin at 37 °C, 5% CO_2_. Soluble tachyzoite antigens (STAg) were prepared from the tachyzoites of ME49 strain with the aid of Ultrasonic disintegrator as previously described [20].

### Construction of recombinant pEGFP-SOD plasmid

**Fig. 1:**
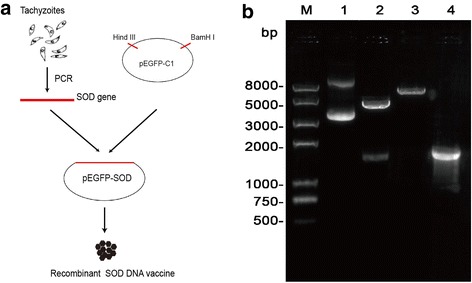
Construction of recombinant plasmid pEGFP-SOD. **a** The schematic diagram of construction of DNA vaccine. **b** Identification of recombinant plasmid pEGFP-SOD using gel electrophoresis analysis. Lane 1, recombinant plasmid pEGFP-SOD; Lane 2, recombinant plasmid pEGFP-SOD was double digested by *Hind* III and *BamH* I enzymes; Lane 3, recombinant plasmid pEGFP-SOD was digested by *BamH* I enzyme; Lane 4, PCR products of SOD gene from pEGFP-SOD; M represents DNA molecular marker

To construct the plasmid expressing recombinant SOD, the SOD gene was amplified by polymerase chain reaction (PCR) from genomic DNA of *T. gondii* tachyzoites of ME49 strain with one pair of primers: 5′- CCC*AAGCTT*ATGGTATTCACTTTGCCCCCGCT-3′ (forward) and 5′- CG*GGATCC*TCATTTCAAGGCATTCTCCAAG-3′ (reverse) containing *Hind* III and *BamH* I sequences (italicized). The amplification reaction was performed under the following program: initial denaturation at 94 °C for 3 min, 30 cycles of denaturation at 94 °C for 30 s, annealing at 63.6 °C for 1 min and extension at 72 °C for 1 min, and final extension at 72 °C for 10 min. Then the PCR product was inserted into the cloning vector pEASY-T1 (Trans Gen biotech Company, China). Subsequently through enzymes digesting and reclaiming, the SOD gene fragment was gained and subcloned into pEGFP-C1 (Clontech Company, USA). Finally, the recombinant plasmid was constructed and named as pEGFP-SOD and the schematic diagram of construction of DNA vaccine was shown in Fig. 1a.

### Expression of TgSOD in vitro

To test the expression of recombinant plasmid in vitro, the recombinant plasmid pEGFP-SOD was transfected into HEK 293 T cells using the LipofectamineTM 2000 reagent (Invitrogen, USA) in 6-well tissue culture plates. The HEK 293 T cells transfected with the empty vector pEGFP-C1 was used as a negative control. Then the transfected cells were incubated at 37 °C, 5% CO_2_ for 48 h. Those cells were observed under a fluorescence microscope (Carl Zeiss, Germany) at 24 h post transfection. To prepare cell lysates for sodium dodecyl sulfate-gel electrophoresis (SDS-PAGE), cells were lysed with RIPA lysis buffer (Solarbio, China) containing 1 mM protease inhibitor PMSF. Equal amount of proteins (30 μg per sample) were loaded onto a SDS-PAGE gel. The proteins were separated following a separation process for 1 h at 100 V. Then the proteins were semi-dry transferred onto nitrocellulose (PVDF) membranes (Millipore, USA) at 80 V for 60 min. Nonspecific binding sites were blocked with 5% skimmed milk powder in TBST (TBS plus 0.1% Tween-20) for 2 h at room temperature (RT). The PVDF membranes were then incubated overnight at 4 °C with the sera of immunized mice on week 8 (diluted in 1:200) and GAPDH (Abbkine, USA; diluted in 1:2000) as internal reference. After being washed 3 times with TBST, the membranes were incubated with diluted secondary antibody (goat anti-mouse IgG-HRP, Proteintech, USA; 1:5000) for 2 h at RT. After through washing, the membrane was soaked in DAB Reagents (Millipore, USA) for signal development and was imaged using ChemiScope6300 (Clinx Science Instruments, China).

### DNA vaccine immunization in BALB/c mice and parasite challenge

BALB/c mice were randomly divided into four groups (12 mice per group). Before inoculation, plasmids were diluted and suspended in sterile phosphate buffered saline (PBS) to a final concentration of 1 μg/μl. The 100 μl pEGFP-SOD was used to immunize the mice for the experimental group. As negative controls, groups of mice were either untreated or injected with 100 μl pEGFP-C1 or 100 μl sterile PBS. All groups of mice were immunized in the same manner three times on days 1, 15 and 29, respectively. Four weeks after the last immunization, 9 mice from each group were randomly chosen and put into infection experiments that each mouse was injected intraperitoneally with a dose of 1 × 10^3^ tachyzoites of *T. gondii* ME49 strain. The rest mice (3 mice per group) were used for removing spleen under sodium pentobarbital anaesthesia and for further determination of cytokine production.

### Antibody determination assays

Blood samples of mice in each group were collected by retro-orbital plexus puncture on days 0, 14, 28 post immunization and four weeks after the final immunization (on day 56), then centrifuged at 5000×g for 10 min, and the sera isolated were stored at −20 °C for further serological analysis. The levels of antigen-specific IgG and IgG subclasses in mouse sera were determined by enzyme-linked immunosorbent assays (ELISA). Briefly, STAg of ME49 strain was diluted to 12.5 μg/ml with coating buffer (pH 9.6), and divided into 96-well enzyme panel with 50 μl per well overnight at 4 °C. Then, the plates were blocked with 5% bovine serum albumin (Solarbio, China) at 37 °C for 2 h. The plates were washed three times with PBST, 50 μl diluted mouse serum samples (serum: diluent = 1: 100) were added to the wells and incubated at 37 °C for 1 h with gentle shaking. After washing three times with PBST, 100 μl diluted enzyme-labeled second antibody (IgG-HRP, IgG1-HRP or IgG2a-HRP, 1:1000 dilution, Proteintech, USA) was added in each well, incubated at 37 °C for 1 h. The reaction was terminated by addition of 50 μl of stop solution and the optical density was measured at 450 nm using an automated ELISA reader (Allsheng, China). All sera samples were run in triplicate.

### Cytokine assays

The splenocyte suspension was prepared from spleens of each group four weeks after the last immunization (on day 56). Splenocytes were cultured in vitro in the presence of concanavalin A (Con A; 5 μg/mL; Sigma; positive control), STAg (10 μg/mL) or medium alone (negative control), respectively. Culture supernatants were collected at indicated time points. Interleukin-4 (IL-4) levels were measured at 24 h post stimulation, whereas Interleukin-10 (IL-10) and IFN-γ were measured at 72 h and 96 h post stimulation, respectively. Cytokine level was determined by ELISA according to the manufacturer’s instructions (Chengsen Trading Co., China). All assays were performed in triplicate.

### T cell subsets determination

The percentages of T cell subsets CD4^+^ T cells and CD8^+^ T cells in spleen were determined by flow cytometry as previously mentioned [21]. The concentration of splenocyte suspension was adjusted to 1 × 10^7^ cells/ml in 100 μl of PBS, and cell surface was stained with FITC-conjugated anti-mouse CD4 monoclonal antibody (mAb), Cy5.5-conjugated anti-mouse CD8 mAb (eBioscience) and PE-conjugated anti-mouse CD3 mAb for 0.5 h at 4 °C in the dark. Then the stained cells were washed with PBS for 3 times. All samples were collected and analyzed by a BD FACS Calibur (BD Biosciences, USA).

### Statistical analysis

All data in the study, including results of the humoral responses, cytokine production and flow cytometric assays were processed and analyzed by one-way analysis of variance (ANOVA). The survival times for the vaccinated and control groups were checked by Kaplan-Meier method and compared with the log-rank test. The value of *p* < 0.05 was considered to be statistically significant.

## Results

### Identification of recombinant plasmid pEGFP-SOD

SOD gene was subcloned into the eukaryotic expression vector pEGFP-C1 (pEGFP-SOD). In order to ensure the correct insert orientation, it was tested by PCR and endonuclease digestion. As shown in Fig. 1b, the SOD gene was a specific, approximate 1700 bp in length on agarose gel, which was characterized by restriction endonuclease digestion as well as PCR amplification. Furthermore, DNA sequencing result showed that the SOD gene was 1706 bp with 100% identity to *T. gondii* ME49 strain (not shown here), suggesting that the recombinant plasmid pEGFP-SOD was correctly constructed.

### Identification of expression protein in vitro

**Fig. 2:**
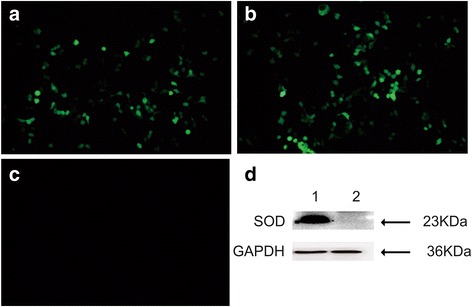
Identification of TgSOD expression in vitro by fluorescence microscopic detection and Western blotting. Fluorescence microscopy images of TgSOD protein in (**a**) HEK 293 T cells that were transfected with pEGFP-SOD and (**b**) empty plasmid pEGFP-C1, and (**c**) non-transfected HEK 293 T cells; (**d**) Western blotting of pEGFP-SOD expressed in HEK 293 T cells (lane 1) probed with anti-STAg mouse sera as primary antibody and the protein of SOD is 23 KDa, whereas no band in the negative control cells with the empty plasmid pEGFP-C1 (lane 2) and GAPDH serves as a loading control

To test the expression of SOD from the constructed plasmid, the pEGFP-SOD was transfected into HEK 293 T cells. The specific green fluorescence was observed in the HEK 293 T cells transfected with the recombinant plasmid pEGFP-SOD (Fig. 2a, b), but not in the untrasfected cells (Fig. 2c), showing that recombinant was expressed efficiently in eukaryotic cells. Western blotting demonstrated that the expressed protein (lane 1) were reacted with anti-STAg mouse sera, whereas the control empty plasmid-transfected cells did not show any band (lane 2) upon incubation with the same antibody (Fig. 2d). The results showed that the protein of SOD was approximately 23 kDa, which was consistent to the expected molecular weight [18], and was recognized by anti-STAg mouse sera as antibody.

### Humoral immune responses

**Fig. 3:**
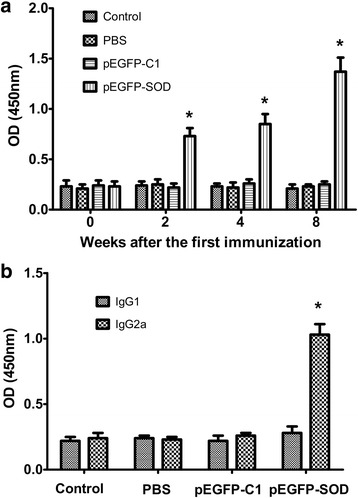
*Toxoplasma*-specific antibody levels in the sera of immunized BALB/c mice. The total IgG antibodies (**a**) in the collected serum samples of BALB/c mice immunized with pEGFP-SOD, pEGFP-C1, PBS and blank control on weeks 0, 2, 4, 8 were analyzed by ELISA. The levels of IgG1 and IgG2a (**b**) subtypes in the sera 28 days after the last immunization were determined by ELISA. The results are expressed as the means ± SD *from three independent experiments*. *p* < 0.05, pEGFP-SOD vs controls

To evaluate the levels of antibody in sera from the pEGFP-SOD group and the control groups, the total IgG was detected after three consecutive DNA immunizations (on weeks 0, 2, 4, 8). As shown in Fig. 3a, the level of IgG detected in pEGFP-SOD group was significantly higher (*p* < 0.05) than them of control groups. Moreover, with the increase of immunization frequency, the IgG level in serum from pEGFP-SOD group increased, it reached the peak in the fourth week after the final immunization.

IgG subtype, IgG1 and IgG2α, in each serum sample were measured by ELISA (on day 56). As presented in Fig. 3b, in immunized group, the levels of IgG1 and IgG2α were obviously higher than that in control groups. The percentage of IgG2α and IgG1 in the pEGFP-SOD group was the highest among all the groups with the dominance of IgG2α over IgG1, whereas IgG1 level showed no significant difference, suggesting that pEGFP-SOD could elicit a Th1 type cellular immune response.

### Cytokines production in vitro

To determine the cytokine response in mice immunized with the vaccine pEGFP- SOD, the levels of IL-4, IL-10 and IFN-γ that were secreted by spleen cells from all remaining immunized mice were measured by ELISA. The results in Table 1 showed that significantly higher level of IFN-γ was observed in the pEGFP-SOD group compared with that in blank control, PBS- and pEGFP-C1-immunized mice groups (*p* < 0.05). However, the levels of IL-4 and IL-10 among all of the study groups displayed no statistically differences.

**Table 1:** Cytokine concentrations of splenocyte supernatants from the immunized BALB/c mice (*n* = 3 animals per group)

Groups	Production of cytokine (pg/mL)		
	IFN-γ	IL-4	IL-10
Blank control	129.30 ± 37.82	22.45 ± 7.22	58.66 ± 11.49
PBS	119.36 ± 34.77	19.53 ± 6.03	61.31 ± 15.40
pEGFP-C1	125.42 ± 41.29	20.65 ± 8.63	64.36 ± 16.93
pEGFP-SOD	3124.55 ± 242.20*	24.96 ± 11.48	62.57 ± 16.26

Cytokine production from splenocytes after stimulations in vitro. Results are expressed as means ±SD from three independent experiments. IFN-γ levels were measured at 96 h post stimulation; IL-10 and IL-4 were measured at 72 h and 24 h post stimulation, respectively

*Compared with blank control, PBS or pEGFP-C1 controls, *p* < 0.05

### Analysis of T cell subsets

As demonstrated in Table 2, in experimental groups, the percentage of CD4^+^ T cells showed somewhat statistical difference with that in control groups. However, between the experimental groups and the control groups, the obvious difference was found in the percentage of CD8^+^ T cells. As for CD8^+^ T cells, the pEGFP-SOD group showed the highest proportion (*p* < 0.05) four weeks after the last vaccination compared with the control groups (Blank control, PBS or pEGFP-C1 controls).

**Table 2:** Percentages of CD4^+^ T cells and CD8^+^ T cells subsets in immunized BALB/c mice (*n* = 3 animals per group) by flow cytometry

Groups	CD3^+^ CD4^+^ CD8^−^ (%)	CD3^+^ CD8^+^ CD4^−^ (%)
Blank control	19.06 ± 1.89	14.13 ± 1.34
PBS	19.23 ± 1.45	13.08 ± 1.18
pEGFP-C1	18.52 ± 1.36	14.05 ± 1.02
pEGFP-SOD	28.79 ± 2.74*	30.94 ± 3.27*

Results are expressed as the mean ± SD (*n* = 3 animals per group) from three independent experiments. The splenocyte suspensions were detected by flow cytometry

*Compared with blank control, PBS or pEGFP-C1 controls, *p* < 0.05

### Protection against challenge with *T. gondii* ME49 strain in mice

**Fig. 4:**
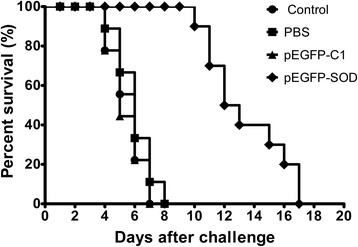
Survival curves of mice in pEGFP-SOD and control groups, following challenge with *T. gondii* ME49 strain. Survival time was monitored daily after challenge with 1 × 10^3^ live tachyzoites of *T. gondii* ME49 strain, 4 weeks after the last immunization (*n* = 9 animals per group). Comparisons of differences between experimental group pEGFP-SOD and control groups (Blank control, PBS or pEGFP-C1) were significant (*p* < 0.05)

The survival curves of the four mice groups after lethal challenge with the *T. gondii* ME49 strain were shown in Fig. 4. The mice immunized with pEGFP-SOD had a significant increase in the survival days (median survival of 12.5 days) in comparison to the control mice unvaccinated, injected with PBS and pEGFP-C1 (median survival of 6, 6 and 5 days respectively). Although all the immunized mice died within 17 days and all the control mice died within 4–8 days post challenge, the results demonstrated that the immunized mice group with pEGFP-SOD had significantly prolonged survival rates as compared to the controls (Blank control, PBS and pEFGP-C1) (*p* < 0.05).

## Discussion

*T. gondii* is one of the most important opportunistic Apicomplexan parasites, whereas no available drugs could effectively eliminate the pathogen from the host [1–3]. In recent studies, DNA-based vaccination has been considered to be a promising approach for the development of vaccine since it could protect animals and humans from viral, bacterial, and even intracellular parasitic infection, including intracellular parasites [22]. Owing to easy production and low cost of DNA vaccines, together with the fact that DNA vaccine could elicit long-lasting humoral and cellular immune responses [22, 23], the development of effective DNA vaccines has emerged as an active research field in the fight against toxoplasmosis [24]. And the use of a single antigen as DNA vaccines against experimental toxoplasmosis was especially investigated in the last few years [25–28]. In the present study, a DNA vaccine encoding *T. gondii* SOD was constructed and the SOD protein was expressed in HEK 293 T cells in vitro. The SOD was identified to play a role in affecting the intracellular growth of both bradyzoite and tachyzoite forms in *T. gondii*. Our present results exhibited that the DNA vaccine encoding *T. gondii* SOD could partially protect the challenged BALB/c mice with a lethal dose of *T. gondii* ME49 strain through inducing highly significant cellular and humoral immune responses, illustrating SOD can be an alternative vaccine antigen for preventing *T. gondii* infection.

Given the features of *T. gondii* being an intracellular parasite, specific cellular immune response, particularly, a specific T lymphocytes activation (CD4^+^ T cells and CD8^+^ T cells) can play a vital role in the alleviation or control of spreading and development of *T. gondii* in infection [29]. Both CD4^+^ T cells and CD8^+^ T cells are well-characterized lymphocytes that respond to intracellular pathogens. The CD4^+^ is one of the surface markers of T helper (T_H_) cells that participate in the adaptive immune responses, while CD8^+^ is expressed on cytotoxic T cells (CTLs), which are classified as a pre-defined cytotoxic role in the immune system [30]. CD4^+^ T cells appear critical in limiting pathogen further growth in the early stage of intracellular infection, whereas CD8^+^ T cells are particularly important in the later stage of infection [30]. IFN-γ produced by Toxoplasma-specific CD4^+^ and CD8^+^ T cells further contributes to the host defense against *T. gondii* infection [31]. The production of IFN-γ in response to intracellular microbial exposure is critical to indicate the partial immunity against *T. gondii* by controlling the growth of the parasite in the acute phase [32]. IFN-γ also plays a vital role in promoting naive CD4^+^ T cells (Th0 cells) to differentiate into Th1 cells, while restraining from Th2 cell differentiation [33]. IL-4 and IL-10 that are produced by CD4 ^+^ Th2 cells are two anti-inflammatory cytokines, which have a significant function to balance and alleviate the harmful inflammatory effect, and favor a Th1 immune response for the control of *T. gondii* infection [34]. In the present study, the mice immunized with pEGFP-SOD were observed to produce higher percentage of CD8^+^ and CD4^+^ T cells, especially the CD8^+^ T cells (*p* < 0.05). Additionally, higher levels of IFN-γ were induced in mice from pEGFP-SOD group compared to the control groups. In contrast, the SOD DNA vaccine failed to stimulate the production of IL-4 and IL-10 (*p* > 0.05). The data above demonstrated that the immunized mice with pEGFP-SOD vaccine could resist *T. gondii* infection through eliciting Th1 immune response characterized by high levels of IFN-γ, and low levels of IL-4 or IL-10.

Along with cellular immune responses, humoral immunity with specific antibodies also plays an important role in controlling *T. gondii* infection [35]. The specific antibody against *T. gondii* has been confirmed to mainly interfere in the attachment between the parasite and the host cell receptors, and to be involved in the process of killing intracellular parasites by macrophages [36]. In the present study, the results showed that total IgG antibody was induced higher in the experimental group compared with the control groups. Additionally, the IgG subclass (IgG1 and IgG2a) results in the experimental group revealed that IgG2a has an obvious advantage over IgG1. Generally, the production of IgG1 is associated with Th2 type immunity, while IgG2a is closely related to Th1 response [37]. Thus, the high levels of IgG2a from the immunized mice serum in this study evinced that the SOD DNA vaccine could produce Th1 immune response.

Similarly, SOD vaccine has an ability to stimulate protective immune responses against other parasites [38, 39]. For example, the pVAX1-SOD DNA partially protected BALB/c mice from the challenge with *Leishmania amazonensis,* which was associated with a mixed immune response including the production of IFN-γ and IL-4 from CD4^+^ and CD8^+^ T lymphocytes [38]. In addition, the rBmEC-SOD vaccine could excite typical Th1 response against infective larvae and microfilariae in jirds with filarial infection [39]. In agreement with those studies, our investigate proved that the DNA vaccine encoding *T. gondii* SOD triggered strong humoral and cellular immune responses, and aroused partial protective immunity against acute *T. gondii* ME49 strain infection in BALB/c mice. From the above the SOD may be a potentially useful alternative to be developed as an effective vaccine for parasite.

In our previous study [20, 21], the protective efficacy of DNA vaccine was evaluated against *T. gondii* RH (type I) strain. However, RH strain is highly virulent pathogenic and lethal to mice, whereas Type II strain is the predominant lineage causing toxoplasmosis in humans [3]. Thus, in this study, we intentionally chose 1 × 10^3^ tachyzoites of moderate virulent *T. gondii* ME49 strain, as one of type II, to investigate the immune protection of SOD DNA vaccine against challenge acute infection. The survival assay demonstrated that the immunization with SOD DNA vaccine significantly prolonged the survival time of challenged mice in comparison with those of control groups, which indicated that the immunization with SOD DNA vaccine was able to produce a certain level of effective immunity against acute *T. gondii* infection in BALB/c mice model. Unfortunately, the symptoms of the challenged mice in later infection stage and the final death confirmed that the SOD DNA vaccine elicited incomplete protective efficacy, which could be largely attributed to the inappropriate immunization strategy, challenge protocol, and insufficiency evaluation criterion. In addition, mouse strains including the age, gender and weight, parasite strains including the dosage, route and stage, are important factors that might hinder the accuracy of experimental results. Nevertheless, these issues should be considered and explored in the future study.

## Conclusion

In brief, our study demonstrated that the DNA vaccine encoding SOD could trigger strong humoral and cellular immune responses, and elicit partial protective immunity against acute *T. gondii* infection in the murine model. Although SOD elicited only partial protection against acute toxoplasmosis, SOD could be a potential vaccine candidate for further investigations in evaluating the immunogenicity and protective potency of SOD based vaccines against toxoplasmosis.

## Abbreviations

Con A: concanavalin A
ELISA: Enzyme-linked immunosorbent assay
SOD: Superoxide dismutase
STAg: Soluble tachyzoite antigens
TgSOD: Superoxide dismutase of *T. gondii*
